# Evaluation of bioactive sphingolipids in 4-HPR-resistant leukemia cells

**DOI:** 10.1186/1471-2407-11-477

**Published:** 2011-11-07

**Authors:** Aintzane Apraiz, Jolanta K Idkowiak-Baldys, María Dolores Boyano, Gorka Pérez-Yarza, Yusuf A Hannun, Aintzane Asumendi

**Affiliations:** 1 Department of Cell Biology and Histology, School of Medicine and Dentistry, University of the Basque Country, Barrio Sarriena s/n, 48940 Leioa (Bizkaia), Spain; 2 Department of Biochemistry and Molecular Biology, Medical University of South Carolina, 173 Ashley Avenue, 250509 Charleston, SC, USA

## Abstract

**Background:**

*N*-(4-hydroxyphenyl)retinamide (4-HPR, fenretinide) is a synthetic retinoid with potent pro-apoptotic activity against several types of cancer, but little is known regarding mechanisms leading to chemoresistance. Ceramide and, more recently, other sphingolipid species (e.g., dihydroceramide and dihydrosphingosine) have been implicated in 4-HPR-mediated tumor cell death. Because sphingolipid metabolism has been reported to be altered in drug-resistant tumor cells, we studied the implication of sphingolipids in acquired resistance to 4-HPR based on an acute lymphoblastic leukemia model.

**Methods:**

CCRF-CEM cell lines resistant to 4-HPR were obtained by gradual selection. Endogenous sphingolipid profiles and in situ enzymatic activities were determined by LC/MS, and resistance to 4-HPR or to alternative treatments was measured using the XTT viability assay and annexin V-FITC/propidium iodide labeling.

**Results:**

No major crossresistance was observed against other antitumoral compounds (i.e. paclitaxel, cisplatin, doxorubicin hydrochloride) or agents (i.e. ultra violet C, hydrogen peroxide) also described as sphingolipid modulators. CCRF-CEM cell lines resistant to 4-HPR exhibited a distinctive endogenous sphingolipid profile that correlated with inhibition of dihydroceramide desaturase. Cells maintained acquired resistance to 4-HPR after the removal of 4-HPR though the sphingolipid profile returned to control levels. On the other hand, combined treatment with sphingosine kinase inhibitors (unnatural (dihydro)sphingosines ((dh)Sph)) and glucosylceramide synthase inhibitor (PPMP) in the presence or absence of 4-HPR increased cellular (dh)Sph (but not ceramide) levels and were highly toxic for both parental and resistant cells.

**Conclusions:**

In the leukemia model, acquired resistance to 4-HPR is selective and persists in the absence of sphingolipid profile alteration. Therapeutically, the data demonstrate that alternative sphingolipid-modulating antitumoral strategies are suitable for both 4-HPR-resistant and sensitive leukemia cells. Thus, whereas sphingolipids may not be critical for maintaining resistance to 4-HPR, manipulation of cytotoxic sphingolipids should be considered a viable approach for overcoming resistance.

## Background

The synthetic retinoid 4-HPR has potential as a promising chemotherapeutic drug due to its strong pro-apoptotic effect on a variety of tumors, especially on acute lymphoblastic leukemia (ALL) cell lines [1,2]. Thus, 4-HPR is currently being applied in several clinical trials against different tumors [3,4] and has been shown to overcome tumor resistance to ATRA [5]. However, studies in human ovarian carcinoma cell lines [6-8] have shown that resistance to 4-HPR may also develop. Resistance (intrinsic or acquired) to the chemotherapeutic drug remains the main source of failure in current chemotherapeutic treatments [9]. Acquired resistance is an especially complicated problem due to the fact that tumors often not only become resistant to the drug they were treated with, but also to other drugs (multidrug resistance (MDR) phenotype). Thus, determining resistance-related molecular mechanisms is crucial for improving treatment effectiveness.

4-HPR is a well-studied antitumor agent with a mechanism of action that has been linked to oxidative stress induction [2,10,11], as well as to modifications of endogenous sphingolipid (SL) levels [1,8,12,13]. Regarding 4-HPR resistance, studies mentioned above revealed SL profiles as one of the main distinguishing characteristics between 4-HPR-sensitive and 4-HPR-resistant ovarian carcinoma cell lines.

Sphingolipids are lipids based on sphinganine or sphingosine that form a large family of molecules with both structural and signaling functions [14]. According to Prinetti et al. [7] and Maurer et al. [15], 4-HPR increases endogenous ceramide (Cer) levels but induces lower or no Cer accumulation in resistant cancer cells. Combination of 4-HPR with modulators of ceramide metabolism (e.g., inhibitors of Cer conversion into Sph1P or into glucosylceramides) has been proposed as an alternative to increase 4-HPR mediated cytotoxicity [1,8,15,16] or to overcome 4-HPR resistance in ovarian carcinoma models [8,15,17]. On the other hand, glucosylceramide synthase (GCS) activity has been linked to MDR phenotype and P-gp expression [18], suggesting positive feedback among ceramide glucosylation and MDR development. In addition, recent data show accumulation of dihydroceramide (dhCer), and not Cer, upon 4-HPR treatment [8,19]. DhCer was thought for a long time to be the inactive precursor of Cer [20], and is now being implicated in several biological processes, including cell cycle arrest [19] and autophagy [21].

Focusing on the MDR phenotype, no cross-resistance to other natural or synthetic retinoids have been observed in 4-HPR-resistant A2780 cells [6]. Cross-resistance to other SL modulating agents has not been tested. This study is the first to evaluate acquired 4-HPR resistance in ALL and is an in depth analysis of the role of SLs in the 4-HPR-resistance phenotype. The results show that dhCer accumulation, as well as other changes in SL profiles, are reversible phenomena that may be independent of the acquired resistance. Regarding therapeutics, the data reveal that 4-HPR resistance in ALL cells does not follow the MDR phenotype. Furthermore, resistance to this synthetic retinoid can be overcome by manipulating levels of cytotoxic SLs, creating opportunities for other alternative treatments.

## Methods

### Reagents

RPMI 1640 (#11835-034), red phenol free RPMI 1640 (#11835-063), and heat inactivated fetal bovine serum (FBS) (#10082-174) were purchased from Gibco/BRL (Invitrogen). L-glutamine (#G7513), propidium iodide (PI), 5-bromo-2-deoxyuridine (BrdU), 4-(hydroxyphenyl)retinamide (4-HPR), d,l-*threo*-1-phenyl-2-palmitoylamino-3-morpholino-1-propanol (PPMP), d,l-*threo*-dihydrosphingosine (DHS), 4-[[4-(4-chlorophenyl)-2-thiazolyl]amino]phenol (SKI-II), paclitaxel, doxorubicin hydrochloride (Adriamycin^® ^hydrochloride), *cis*-diammineplatinum(II) dichloride (cisplatin), and H_2_O_2 _were purchased in Sigma Chemical Co. L-*threo*-dihydrosphingosine (safingol), D-*erythro*-sphinganine (C17 base) (C17-dhSph), and D-*erythro*-sphingosine (C17 base) (C17-Sph) were purchased from Avanti Polar Lipids, Inc. The cell viability XTT assay kit (#11465015001) was purchased from Roche Molecular Biochemicals and Annexin V-FITC Apoptosis Detection Kit from Calbiochem^®^. D-*erythro*-2-*N*-[12_-(1_-D-*erythro*-2-*N*-[12_-(1_-pyridinium)dodecanoyl]-4,5-dihydrosphingosinebromide (D-*erythro*-C12-dihydroceramide; C_12_-PyrdhCer) was synthesized by the Lipidomics Core Facility at the Medical University of South Carolina [22]. Anti-BrdU-FITC (#347583) was purchased from Becton Dickinson and carboxyfluorescein diacetate succimidyl ester (CFSE; #C34554) from Molecular Probes.

Cells were exposed to a final DMSO concentration of ≤ 0.1%.

### Cell lines and culture conditions

Human CCRF-CEM and Jurkat acute lymphoblastic leukemia cells were purchased from ATCC and cultured in RPMI 1640 with 2 mM L-glutamine supplemented with 10% heat inactivated FBS (complete culture medium). Cells were kept at 37°C in a humidified incubator containing 5% CO_2_. To develop 4-HPR-resistant cells, CCRF-CEM cells were continuously exposed to increasing 4-HPR concentrations, starting from 0.5 μM and up to 10 μM. 4-HPR-containing complete culture medium was replaced every 48-72 h and cells seeded at 0.4 × 10^6 ^cells/ml. Cells were exposed to a higher 4-HPR concentration when death in cell culture was < 20% according to trypan blue exclusion.

### Treatment preparation

4-HPR (#H7779) was dissolved in DMSO to 10 mM, aliquoted, and stored at -80°C. PPMP (#P4191; 1:1 ethanol/H_2_O, 20 mM stock), DHS (#D7033; DMSO/ethanol, 15 mM stock), safingol (#860488P; ethanol 95%, 10 mM stock), SKI-II (#S5696; DMSO, 50 mM stock), paclitaxel (#T7191; DMSO, 10 mM stock), and doxorubicin hydrochloride (#D1515; DMSO, 20 mM stock) were stored at -20°C. Cisplatin (#P4394) and H_2_O_2 _(#H1009) solution were prepared fresh for each experiment. Cisplatin was prepared by dissolving in H_2_O, vortexing 1 min, and shaking for 10 min, followed by incubation at 37°C for 15 min, vortexing, and adding more H_2_O to a final concentration of 400 μg/ml. For ultraviolet (UV) radiation, we used a GS Gene Linker UV chamber (Bio-Rad), which emits UVC light (λ_avg _= 254 nm) at selected doses of 1, 3, or 5 mJ/cm^2^. For UV radiation, cells were plated in 60-mm dishes (approximately 500,000 cells/ml; 5 × 10^6 ^cells/sample) in red phenol-free culture medium.

### Cell viability

Standard XTT assay was used to determine cell viability by means of metabolic activity [23]. Cells were plated in 96-well plates at 750,000 cells/ml and 100 μl/well. After 4 h, treatments were added as 50 μl/well with a final density of 500,000 cells/ml and final volume of 150 μl/well. Four replicates were used per experimental condition, and all compounds were added at the same time. The XTT reagent mixture was added 4 h before the selected endpoint and absorbance at 490 nm determined for each well according to the manufacturer's instructions. Culture media plus XTT reagent was used as the control and the effect of treatments calculated as the absorbance value percentage of corresponding control cells. A slightly modified protocol was used for simultaneous analysis of viability and SL profile by LC/MS and for ultraviolet radiation (UV) treatment. Briefly, cells (approximately 500,000 cells/ml; 5 × 10^6 ^cells/sample) were seeded on 60-mm culture dishes and treatment added after 4 h. For viability studies, aliquots of cells were taken from the 60-mm dish cultures and placed in 96-well plates. Any remaining cells were collected for LC-MS analysis when required. Red phenol-free culture medium was used for UV treatment.

### Cell proliferation (BrdU labeling)

Cells (2 ml/well, 10^6 ^cells/ml) were seeded in 6-well plates in complete culture medium. After 4 h, BrdU (#B5002; 10 μM final concentration) was added to the medium and the cells incubated for 3 h at 37°C and 5% CO_2_. Cells were washed twice with 1% BSA/PBS and re-suspended in 200 μl cold PBS. Cells were fixed with cold 70% ethanol (vortex gently, incubate for 30 min on ice), centrifuged (10 min, 500 g, room temperature), and the pellets vortexed gently and re-suspended in 1 ml 2N HCl/0.5% Triton X-100 (30 min incubation at room temperature) to denature the DNA. After 30 min, cells were centrifuged (10 min, 500 g) and 1 ml Na_2_B_4_O_7_·10H_2_O (pH 8.5) added to the pellets. Samples were centrifuged again and the pellets re-suspended in 1 ml 0.5% Tween20 in 1% BSA/PBS. Anti-BrdU-FITC (20 μl) was added to each sample (10^6 ^cells) and incubated for 30 min at room temperature. After discharging antibody, cells were washed once with 0.5% Tween20 in 1% BSA/PBS, centrifuged, and the pellets re-suspended in 1 ml PBS containing 5 μg/ml PI (#P4170). Cells were incubated for 30 min at room temperature with PI before flow cytometric analysis. Proliferation was estimated as the percent of FITC-positive cells with 2C (G0/G1) to 4C (G2/M) DNA content and graphically represented as the percent of FITC-positive cells relative to the CCRF-CEM cell line.

### Apoptotic cell death

Apoptotic cell death was analyzed in order to establish cellular sensitivity/resistance to 4-HPR. Cells were treated and cell death determined by annexin V-FITC and PI staining using the Annexin V-FITC Apoptosis Detection Kit (PF032) according to the manufacturer's instructions. Flow cytometric analysis of apoptotic cell death was performed using a Beckman Coulter Gallios cytometer in the General Research Services SGIker of the UPV/EHU (http://www.ikerkuntza.ehu.es/p273-sgikerhm/en/).

### LC/MS analysis of endogenous sphingolipid species

Cells were seeded and treated in 60-mm culture dishes as described above. After the selected treatment time, cells were washed twice with PBS to avoid external SL contamination from media and further analysis performed by the Lipidomics Core Facility of the Medical University of South Carolina (http://www.musc.edu/BCMB/lipidomics/index.shtml) as described previously [22,24]. Total values were normalized to endogenous inorganic phosphates (Pi) using adapted Bligh and Dyer lipid extraction [25].

### *In situ *dihydroceramide desaturase assay

C_12_-PyrdhCer was used as a substrate for the dihydroceramide desaturase enzyme. The synthetic analogue was dissolved in 100% ethanol at a stock concentration of 100 mM and stored at -20°C, protected from light. Aliquots of the stock solution were diluted and added to cells in complete culture medium. The final C_12_-PyrdhCer concentration in medium was 500 nM. When required, C_12_-PyrdhCer was added together with 4-HPR and incubated for the selected time points. Levels of C_12_-PyrdhCer and its product (C_12_-PyrCer) were analyzed by LC/MS as described previously [22,24].

### *In situ *estimation of dihydrosphingosine and sphingosine utilization

Estimation was determined by the utilization of unnatural dihydrosphingosine (C17-dhSph; #860654P) or sphingosine (C17-Sph; #860640P) species as previously described by Spassieva et al. [26]. Cells were seeded and treated in 60-mm culture dishes as described above and C17-dhSph or C17-Sph (500 nM final concentration; 10 mM stock solution in ethanol, -20°C) incubated for 30 min prior to ending the defined treatment. Cells were collected on ice, washed with cold PBS, and analyzed by LC/MS. Data were normalized to Pi content and calculated as the percent of product (selected C17 species) in relation to total C17 species in the cell.

### Statistical analysis

Results are expressed as the mean ± standard deviation (SD). The number of replicates is specified for each experiment. The significance of normally distributed data (Kolmogorov-Smirnoff test) was determined using analysis of variance (ANOVA) plus Bonferroni or Tamhane post-hoc tests for multiple comparisons. Bonferroni or Tamhane was chosen based on the equality of variance in samples (Levene's test). The Kruskal Wallis test with Mann-Whitney test for pair-wise comparison was used for data that was not normally distributed (SPSS, version 15.0). Significance was set at *P *< 0.05 or *P *< 0.01 as specified for each figure.

## Results

### Continuous exposure to increasing 4-HPR concentrations leads to acquired drug resistance phenotype

**Figure 1:**
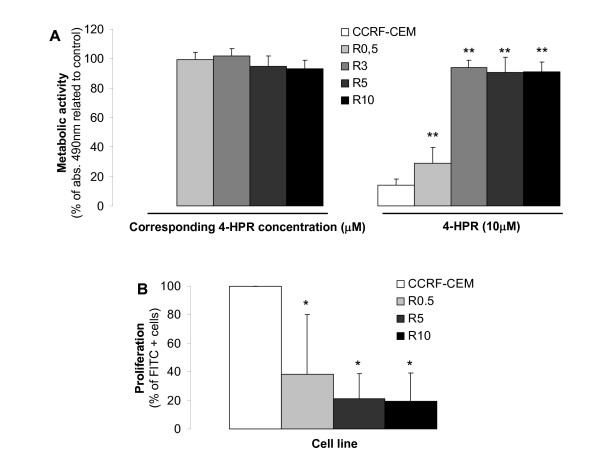
**Acquired resistance upon long-term 4-HPR exposure**. (A, left) R cell lines were continuously exposed to different 4-HPR concentrations (0.5, 3, 5, and 10 μM; cell lines named R0.5, R3, R5, and R10, respectively) and resistance verified by comparison to 48 h drug-withdrawn R cells. Data are the average ± SD of three independent experiments performed in quadruplicates, n = 12. (A, right) Parental CCRF-CEM cells and developed R cell lines were exposed to 10 μM 4-HPR and viability estimated after 48 h of drug exposure. (n = 12). **P < 0.01; ANOVA plus Tamhane post-hoc analysis. (B) Comparative cell proliferation was determined by BrdU labeling as specified in Materials and Methods. Data are average ± SD of four independent experiments. *P < 0.05; Kruskal Wallis and Mann-Whitney test for pair-wise comparison.

Resistance in 4-HPR-resistant CCRF-CEM cell lines (R cell lines) was determined by XTT assay upon exposure to the drug for 48 h. R cell lines selected at fixed concentrations of 4-HPR (R0.5, R3, R5, and R10) became fully resistant to the corresponding 4-HPR concentrations (i.e. 0.5 μM, 3 μM, 5 μM and 10 μM; Figure 1A). Moreover, R cell lines developed partial (R0.5) or full (R3, R5) resistance to 4-HPR concentrations up to 10 μM. Thus, we were able to develop cell lines resistant to various concentrations of 4-HPR.

Interestingly, all R cell lines had a significantly lower proliferation rate than parental CCRF-CEM cells (*P *< 0.05) without significant differences among R cell lines based on BrdU incorporation rate (Figure 1B). These results indicate changes not only in cell survival, but also cell cycle progression.

### Acquired resistance against 4-HPR does not fulfill multidrug-resistance phenotype

**Figure 2:**
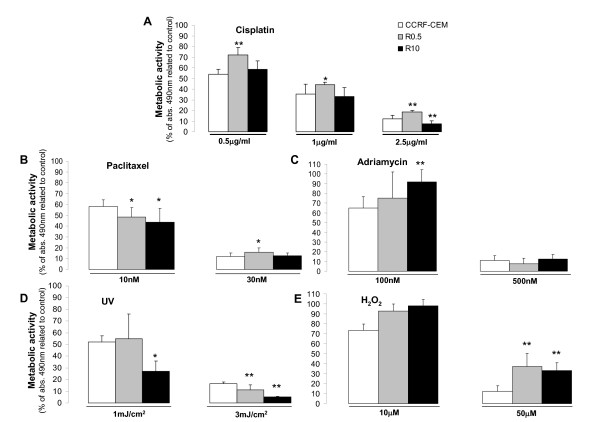
**Effect of selected insults on the viability of 4-HPR-sensitive and resistant leukemia cell lines**. 4-HPR-sensitive (CCRF-CEM) and derived resistant cell lines (R0.5 and R10) were exposed to several drugs or UV radiation, and cytotoxicity tested after 48 h by the XTT assay. Data are average ± SD of three independent experiments performed in quadruplicates (n = 12); *P < 0.05, **P < 0.01; ANOVA plus Tamhane post-hoc test.

4-HPR-sensitive parental leukemia cell line (CCRF-CEM) and 4-HPR-resistant cell lines (R0.5 and R10) were challenged by different anticancer drugs, such as cisplatin (0.5, 1, 2.5, or 5 μg/ml), paclitaxel (1, 5, 10, 30, or 100 nM), or adriamycin (50, 100, or 500 nM) and other insults such as UV irradiation (1, 3, or 5 mJ/cm^2^) or direct oxidative stress (H_2_O_2; _10, 50, or 100 μM). Based on metabolic activity, cell variability was analyzed after 48 h to determine the endpoint comparative cytotoxicity. Figure 2 summarizes the results obtained with the selected concentrations. No significant major cross-resistance was observed against any of the mentioned agents, and cytotoxicity was directly correlated to the concentration of the drugs. Nevertheless, compared to parental CCRF-CEM cells, R0.5 and R10 cell lines were significantly more resistant to H_2_O_2 _(*P *< 0.05)_. _Interestingly, R0.5 cells exhibited slightly increased sensitivity to UV, and R10 cells exhibited significantly increased sensitivity to UV (*P *< 0.05). Dose-dependent minor differences are detailed in Figure 2.

### The sphingolipid profile in R cell lines reflects prolonged inhibition of DES

**Figure 3:**
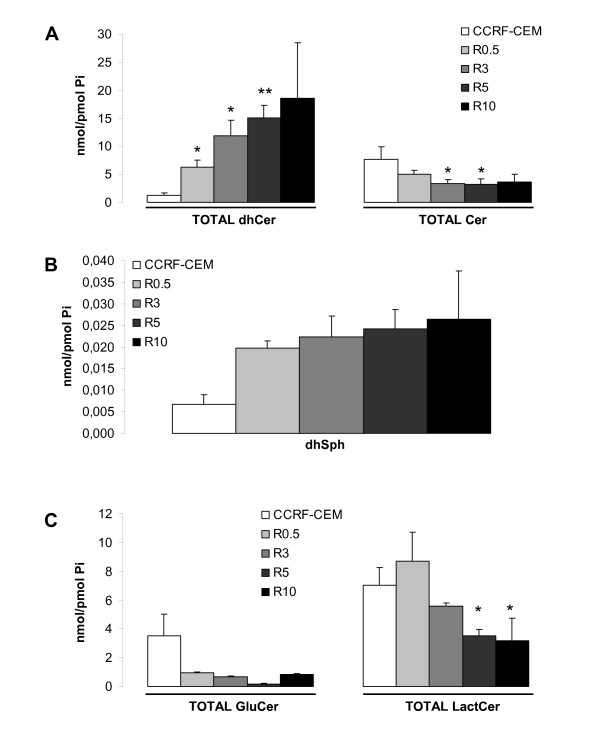
**Comparative endogenous SL pattern in 4-HPR-sensitive and resistant leukemia cell lines**. Cell were grown with (R cell lines) or without (CCRF-CEM) 4-HPR. Endogenous TOTAL (sum of the different fatty acid chain-length species) dhCer (A, left panel) and Cer (A, right panel) levels, as well as dhSph (B), TOTAL GluCer, and LactCer (C) levels were determined by LC/MS. Data are average ± SD of four (A, B) or two (C) independent experiments. *P < 0.05; **P < 0.01; ANOVA plus Tamhane or Bonferroni post-hoc test.

**Figure 4:**
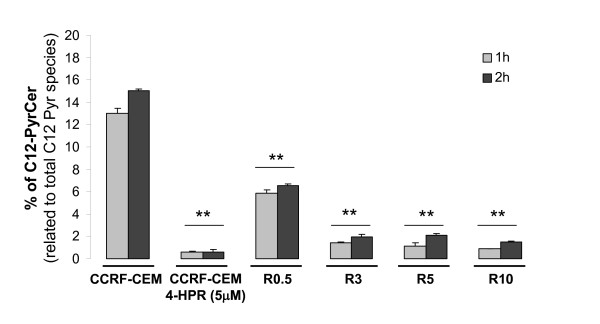
**Comparative analysis of dihydroceramide desaturase (DES) activity**. Cells were incubated with the unnatural pyridinium dhCer (C12-PyrdhCer) alone or with 4-HPR (5 μM) for the selected time points and conversion into C12-PyrCer measured by LC/MS. Data are average ± SD of two independent values. **P < 0.01; ANOVA plus Bonferroni post-hoc test.

LC-MS mediated analysis of the endogenous SL species revealed a clear dose-dependent accumulation of total dhCer and decreased levels of total endogenous Cer in R cell lines compared to parental CCRF-CEM cells (Figure 3A). Also, dhSph (upstream of dhCer on the *de novo *SL synthesis pathway) showed a dose-dependent tendency for accumulation in R cell lines (Figure 3B), whereas values for SL species downstream of Cer (e.g., GluCer and LactCer) were significantly decreased (Figure 3C). These data indicate the accumulation of SL species upstream of dihydroceramide desaturase (DES) activity and decreased SL species downstream of DES activity. Thus, the activity of the enzyme was analyzed next (Figure 4). Cellular DES activity was measured by means of the intracellular desaturation of a synthetic dhCer analog (C12-Pyr-dhCer conversion into C12-Pyr-Cer). Similar to what was observed in CCRF-CEM cells upon acute 4-HPR treatment (1-2 h with 5 μM 4-HPR), DES activity was inhibited in all R cell lines (CCRF-CEM *vs*. R0.5, R3, R5, R10 cell lines, *P *< 0.01). No significant differences were observed in the level of inhibition among R3, R5, and R10 cells, whereas R0.5 cells retained significantly higher DES activity compared to the other R cell lines (R0.5 *vs*. R3, R5, R10; *P *< 0.01; Figure 4). Notably, acute treatment of CCRF-CEM cells with 4-HPR (5 μM) induced nearly complete inhibition of DES *in situ *activity within the first hour (conversion rate (%) of 13.00 ± 0.45 in control *vs*. 0.61 ± 0.03 in 4-HPR-treated CCRF-CEM cells). On the other hand, resistant cells under constant 5 μM 4-HPR exposure (R5 cells) showed a somewhat higher desaturation rate than 5 μM 4-HPR-treated parental CCRF-CEM cells (conversion rate (%) of 0.61 ± 0.17 *vs*. 2.09 ± 0.20 at 2 h), which suggests slight adaptation.

### Withdrawal from 4-HPR exposure reverses endogenous SL levels without substantially affecting cell resistance

**Figure 5:**
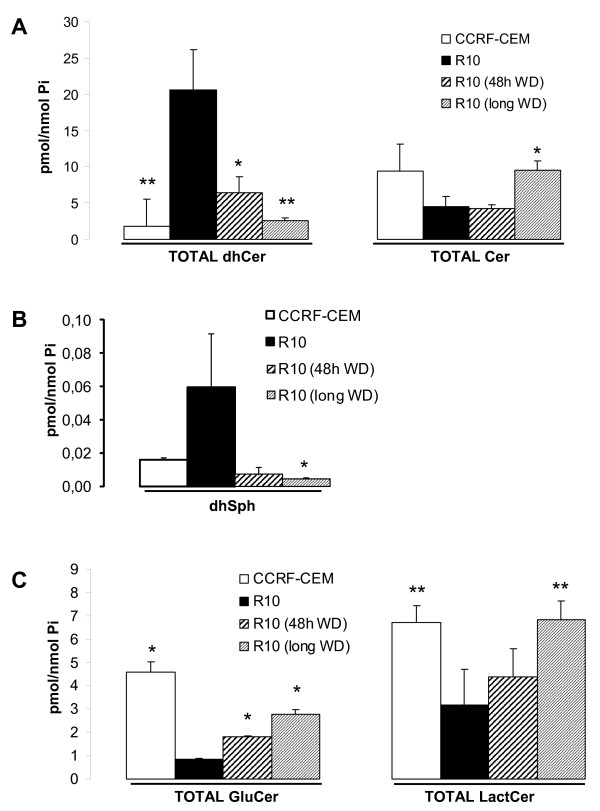
**Comparative SL profile analysis upon drug withdrawal**. R10 cells were incubated in 4-HPR-free medium for 48 h (WD = without drug) or long-term (~3 weeks) and endogenous SL profiles analyzed by LC-MS. Significance was based on R10 values. Data are average ± SD of 2-4 independent values. *P < 0.05; **P < 0.01; ANOVA plus Tamhane or Bonferroni post-hoc test.

**Figure 6:**
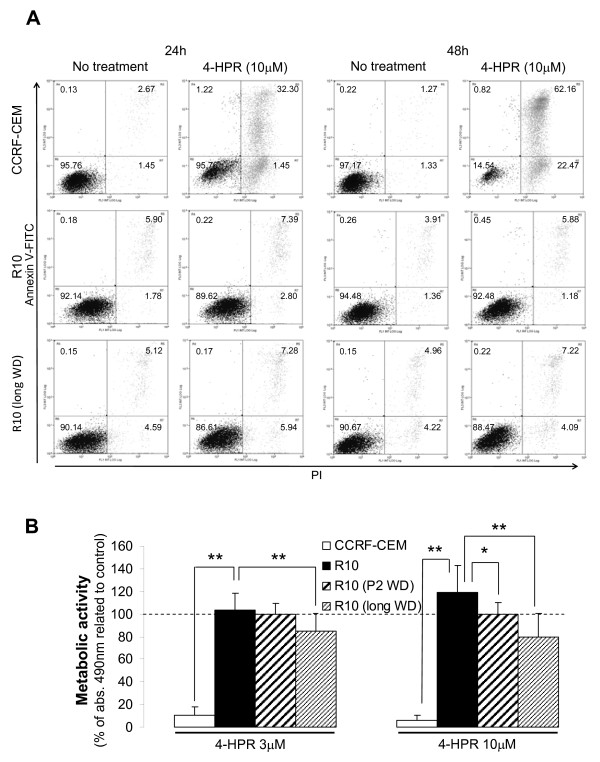
**Effect of drug withdrawal on cell resistance**. (A) Parental sensitive CCRF-CEM cells, resistant R10, and long-term drug withdrawn resistant (R10 longWD) cells were treated and cell death determined by annexin V-FITC/propidium iodide staining at the selected time points. Values represent the percentage of cells in each quadrant. Representative data from two independent experiments are shown. (B) A portion of each sample for SL profile analysis (Figure 5) was saved for XTT assay estimation of resistance. Briefly, R10 cells after 48 h and long-term drug withdrawal were subjected to 48 h treatment with 3 or 10 μM 4-HPR. All data were related to the corresponding untreated cells. Statistical analysis was related to R10 data. Data are average ± SD of 12 values from to three independent experiments. *P < 0.05; **P < 0.01; ANOVA plus Tamhane post-hoc test.

In order to study the reversibility of drug resistance, we removed R10 cells from 4-HPR exposure for either 48 h or long-term (average of 8 passages or 3 weeks) and SL levels analyzed (Figure 5A-C). Resistance was measured simultaneously by annexin V-FITC/PI staining and XTT by challenging drug-withdrawn R10 cells with specific 4-HPR concentrations (Figure 6). Forty-eight hours was enough to alter the endogenous SL profile, resulting in a significant decrease in dhCer levels (Figure 5A) and increased intracellular GluCer (Figure 5C) (R10 vs. R10 48 h WD, *P *< 0.05). Moreover, endogenous dhCer (Figure 5A) and LactCer (Figure 5C) levels after 48 h without 4-HPR were comparable to those obtained in the parental CCRF-CEM cell lines (CCRF-CEM vs. R10 48 h WD; *P *> 0.05). The decrease in dhSph and dhCer levels, as well as the increase in SL species downstream of DES activity (e.g., Cer, GluCer and LactCer), were sustained after long-term absence of 4-HPR in R10 cells (R10 vs. R10 long WD; *P *< 0.05) with all SL values except GluCer being similar to the values in the parental cell line (R10 long WD vs. CCRF-CEM; *P *> 0.05). However, removal of the drug did not reverse the acquired resistance phenotype compared to parental CCRF-CEM cells (Figure 6; CCRF-CEM < R10 48 h WD or CCRF-CEM < R10 long WD; *P *< 0.01), though it did partially affect metabolic activity compared to R10 cell line (R10 > R10 48 h WD or R10 > R10 long WD; *P *< 0.05 or *P *< 0.01 as specified in Figure 6B). Interestingly enough, long-term withdrawal of the drug (R10 long WD) induced not just a recovery of parental (CCRF-CEM) SL levels, but also a recovery of the proliferation rate (additional file 1).

### Sphingolipid modulators as an alternative treatment against 4-HPR-sensitive and resistant leukemia cells

**Figure 7:**
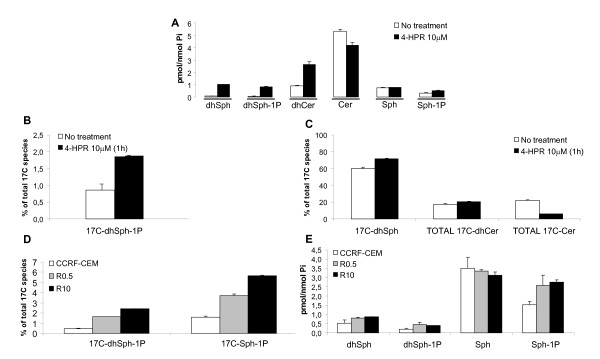
**Analysis of 4-HPR-mediated alterations in dhSph and Sph phosphorylation**. Externally added unnatural dhSph (17C-dhSph) and Sph (17C-Sph) analogues were used to estimate the secondary effects of 4-HPR-induced DES inhibition. CCRF-CEM cells were exposed to 4-HPR (10 μM) for 1 h and unnatural 17C species added for an additional 30 min incubation, as described in Methods. The effect on endogenous SLs (A) and unnatural dhSph species (B,C) analyzed by LC/MS. Comparative exogenous and endogenous SL profiles among parental (CCRF-CEM) and resistant cell lines (R0.5, R10) (D, E) were analyzed by the same methodology. Data are average ± SD of an experiment performed in duplicate.

We have demonstrated that, in resistant leukemia cells, the observed changes in SLs are reversible events distinct from the acquired resistance phenotype. At this point we wanted to address whether increased pro-apoptotic SLs (e.g., Cer [27]) and/or decreased pro-survival species (e.g., dhSph-1P, Sph-1P [27,28]) could enhance 4-HPR-mediated cytotoxicity. First, we studied the changes in phosphorylated SLs upon 4-HPR treatment in parental CCRF-CEM cells. 4-HPR induced a clear increase in dhSph, dhCer, and phosphorylated (dh)Sph species (Figure 7A).

Accumulation in (dh)Sph-1P was likely due to increased (dh)Sph kinase (SK) activity. This possibility was evaluated using 17C-dhSph as a cellular probe to evaluate the activity of sphingosine kinases (Figure 7B,C). One hour pre-treatment with 4-HPR resulted in a 2-fold increase in 17C-dhSph phosphorylation (Figure 7B) and decreased Cer production (17C-Cer) (Figure 7C). Thus, 4-HPR induced flux through sphingosine kinase.

Next, we studied the formation of phosphorylated sphingosine species in 4-HPR-sensitive (CCRF-CEM) and resistant (R0.5-R10) cell lines. After 30 min incubation with 17C-dhSph and 17C-Sph, a clear increase in phosphorylation in resistant cells was observed compared to parental CCRF-CEM cells (Figure 7D). The amount of phosphorylation was proportional to the degree of resistance. Analysis of endogenous SLs confirmed accumulation in dhSph-1P and Sph-1P (Figure 7E). Therefore, 4-HPR resistance is accompanied by increased activity of (or at least flux through) sphingosine kinase.

**Figure 8:**
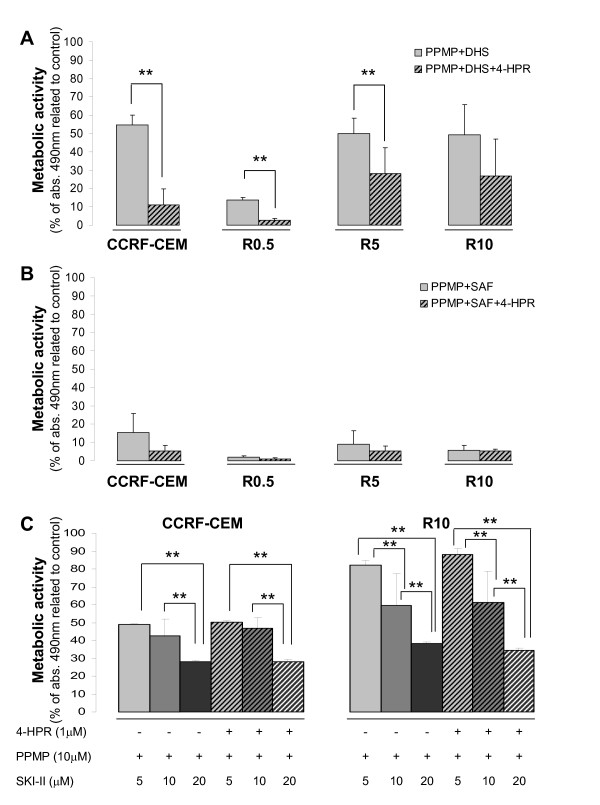
**Toxicity mediated by the combination of 4-HPR with SL modulators**. Parental-sensitive (CCRF-CEM) and derived-resistant (R0.5, R5, R10) cells were co-treated with 4-HPR (1 μM), DHS (5 μM; unnatural dhSph analogue), PPMP (10 μM; glucosylceramide synthase inhibitor), SAF (4 μM; another unnatural dhSph analogue), and/or SKI-II (5-20 μM) and cytotoxicity estimated after 48 h exposure using the XTT assay. Data are average ± SD of at least two independent experiments performed in quadruplicate (n ≥ 8); **P < 0.01; ANOVA plus Tamhane post-hoc test.

Based on the observed decrease in pro-apoptotic Cer levels and increased pro-survival (dh)Sph-1P levels, we combined 4-HPR with PPMP (GCS inhibitor) and the unnatural dhSph, d,l-*threo*-dhSph (DHS; competitive inhibitor of (dh)Sph kinase). The data revealed that, in the parental cell line (CCRF-CEM), the combination of 4-HPR with 5 μM DHS or ≥ 5 μM PPMP increased 4-HPR (1 μM)-mediated cytotoxicity with 24 h exposure (*P *< 0.01; additional file 2A-B). The combination of both SL modulators significantly increased the effects of each agent alone (*P *< 0.01). Thus, a mixture of both SL modulators with 1 μM 4-HPR (complex DHS treatment) resulted in the highest toxicity in parental cells (additional file 2; viability PPMP+DHS (58.91 ± 7.54) *vs*. PPMP+DHS+4-HPR (22.29 ± 16.81); *P *< 0.01). Interestingly, DHS-based treatment induced cytotoxicity not just in 4-HPR-sensitive cells (CCRF-CEM), but also in 4-HPR-resistant cells (R0.5, R5, R10; Figure 8A). Substitution of d,l-*threo*-dhSph (DHS) by l-*threo*-dhSph (safingol) increased the cytotoxic response to combined therapy (PPMP+DHS+4-HPR *vs*. PPMP+SAF+4-HPR), especially in 4-HPR-resistant R5 and R10 cells (*P *< 0.01; Figure 8B). Moreover, combination of PPMP with SAF increased cell toxicity in both 4-HPR-sensitive and resistant cell lines, even in the absence of 4-HPR (PPMP+DHS *vs*. PPMP+SAF; *P *< 0.01). Notably, SAF concentrations > 4 μM were highly toxic (data not shown). SKI-II, a new generation sphingosine kinase inhibitor, corroborated the data obtained with DHS or SAF-based drug combinations (Figure 8C). Therefore, inhibition of selected pro-survival SL pathways represents an alternative antitumor strategy, even in the absence of 4-HPR, and is suitable for both 4-HPR-resistant and sensitive cells.

LC-MS mediated analysis of SLs in 4-HPR-sensitive cell lines (CCRF-CEM, Jurkat) revealed that none of the SL modulators alone or in combination with 4-HPR increased endogenous total Cer levels (additional file 3A-D). As expected, the GCS inhibitor PPMP decreased GluCer levels (additional file 4B) and induced a transient increase in Cer content (additional file 3B). However, the observed effect on Cer levels was not present when PPMP was added with other SL modulators (i.e. combination treatments) (additional file 3D). Unnatural dhSph species, especially SAF, induced an increase in both phosphorylated and non-phosphorylated (dh)Sph species (additional file 5A) and dhCer levels (additional file 3A), as well as a decrease in GluCer species (additional file 4A). Changes driven by unnatural dhSph species were also observed in response to combination treatments (additional file 3D, 4C, 5C). Thus, the selected SL modulators do not act through an early increase in cellular Cer levels, though the previously observed cytotoxicity may involve other sphingoids or later ceramide production.

## Discussion

Tumor cell plasticity may easily lead to secondary drug resistance or the MDR phenotype. 4-HPR, a synthetic derivative of ATRA, has been shown to exert a potent antitumoral effect against several types of tumors [2,12,29]. Nevertheless, resistance to 4-HPR has been observed in human ovarian carcinoma cell lines [6-8]. In this study, we developed and characterized resistance in human ALL cells as an appropriate model due to its remarkable primary sensitivity to 4-HPR [1,2].

4-HPR has been described to induce accumulation of endogenous Cer [1,12,13] based on methods that do not allow differentiation of Cer from its precursor, dhCer (e.g., [^3^H]palmitic acid labeling plus TLC separation). In a previous study, Prinetti et al. [7] showed that 4-HPR did not induce Cer accumulation in 4-HPR-resistant ovarian carcinoma A2780 cells. Notably, the resistant A2780 cells were developed and maintained under sustained 4-HPR exposition as in the leukemia model presented in this study. The current study demonstrates that 4-HPR exerts similar inhibition of DES in both acutely treated CCRF-CEM and long-term treated resistant R cells. Therefore, continuous DES inhibition in R cells induces a clear dose-dependent accumulation of dhCer and dhSph. Diminished radioactive (dh)Cer formation upon [^3^H]sphingosine labeling and 4-HPR treatment in resistant A2780 carcinoma cells [7] could be interpreted as the inability of these cells to metabolize externally added [^3^H]sphingosine due to the pre-existing accumulation of endogenous substrate (dhSph) and product (dhCer) of Cer synthases. The study in ovarian cells detected a significant decrease in LactCer levels, which was also found in our leukemia model and could be explained by the sustained DES inhibition.

Altered endogenous SL profiles in ALL R cells were partially reversed after withdrawing 4-HPR for 48 h and comparable to parental cells after long-term withdrawal. The recovery of normal SL profiles after drug withdrawal did not abolish cell resistance to 4-HPR, as clearly shown by the classical apoptotic cell death analysis in Figure 6A, though long-term withdrawal of the drug slightly altered the metabolic activity of the resistant cells. These data suggest that despite profound, persistent changes in endogenous SL levels are not required for the observed resistance in our leukemia model. Nevertheless, SL roles in the induction or modulation of initial drug resistance cannot be ruled out.

A reduction in the colony-formation capacity was observed in resistant A2780 cells [6], and 4-HPR mediated accumulation of dhCer has been linked to cell cycle arrest [19]. In the 4-HPR resistant leukemia model, we found a significant reduction in cell proliferation with a clear negative correlation between the proliferation rate, DES inhibition level, and consequent dhCer accumulation. On the other hand, our results show that, upon drug withdrawal, decreased endogenous dhCer levels are accompanied by a clear increase in the proliferation rate of resistant cells (additional file 1). In this regard, the data suggest that endogenous dhCer content could play a role in regulating the proliferation of resistant cells.

The results of this study also have implications for the roles of SLs in overcoming resistance to 4-HPR. Combinations of 4-HPR with SL modulators, such as PPMP (GCS synthase/1-ACS inhibitor) or competitive SK inhibitors (e.g., unnatural *threo*-dihydrosphingosines DHS/SAF or sphingosine derivative N,N-dimethylsphingosine) have been described as an alternative for increasing efficacy of 4-HPR-based treatments [1,8,15,16]. Additional data support the application of such drug combinations against drug-resistant cells [8,15]. GCS over-expression and elevated GluCer levels have been related to drug resistance in some cancer cells [30,31]. In leukemia, GCS inhibition has been proposed to reverse resistance to vincristine and/or doxorubicin [32,33] by a mechanism that is probably linked to intracellular Cer accumulation. In contrast, the current data do not show a close relationship between 4-HPR resistance and increased GluCer levels in ALL cells. Both GluCer and LactCer levels were decreased in R cells compared to parental 4-HPR-sensitive CCRF-CEM cells. Moreover, recovery of parental GluCer and LactCer levels did not abolish the resistant phenotype. Focused on 4-HPR, opposing results have been reported regarding the toxicity of tumor cells upon combined treatment with GCS inhibitors [15,34,35]. In ALL cells we showed enhanced cytotoxicity when combining PPMP with 4-HPR (in agreement with O'Donnell et al. [1]) or DHS. However, our SL profiles with combination treatments (PPMP+DHS+4-HPR and PPMP+SAF+4-HPR) suggest that, in contrast to previously reported data, cytotoxicity is not likely to be related to Cer content.

Based on the pro-survival functions described for dhSph-1P and especially for Sph-1P, as well as the pro-apoptotic effect driven by Sph and Cer [36], inhibition of SK seems a logical approach for increasing malignant cell death. Wang et al.[8] showed for the first time that 4-HPR increases dhSph and dhSph-1P in colon adenocarcinoma (HT29), promyelocytic leukemia (HL-60), and multidrug resistant ovarian carcinoma (NCI/ADR-RES) cell lines. We have now shown that the 4-HPR-induced dhSph-1P accumulation in T-ALL cells (CCRF-CEM) is due to augmented dhSph phosphorylation. Moreover, our results prove that, in 4-HPR-resistant cell lines (R0.5-R10), not only dhSph, but also Sph phosphorylation is increased, suggesting higher SK activity, which is in agreement with the results obtained by Illuzzi et al. [17]. In addition, the observed cytotoxicity of DHS, SAF, and SKI-II-based treatments supports the use of SK inhibitors in both 4-HPR-sensitive and resistant leukemia cell lines as previously suggested for ovarian carcinoma cells [8]. DHS and SAF are known not just as SL modulators, but also as protein kinase C (PKC) inhibitors [37,38]. SKI-II has been described as a selective sphingosine kinase inhibitor at concentrations below 60 μM [39] with *in vivo *antitumor activity [40]. The differential cytotoxicity achieved by SAF and SKI-II in Figure 8B-C could be due to the non-specific kinase inhibition driven by SAF. In fact, our trials to mimic PKC inhibition with a pharmacological approach confirmed the susceptibility of ALL cell lines to PKC inhibition (unpublished observation). Nevertheless, the data clearly show the role of sphingosine kinase in tumor cell survival and support its inhibition as an important antitumor strategy.

Regarding other therapeutic alternatives for 4-HPR-resistant cells, data from Appierto et al. [6] show no cross-resistance to the synthetic retinoic CD437. In addition, cells resistant to 4-HPR are sensitive to the 4-HPR metabolite 4-oxo-4HPR [41]. Interestingly, other studies reported that CD437-resistant leukemia and ovarian carcinoma cell lines retained sensitivity to 4-HPR [42,43], suggesting specific (*vs*. general) resistance against retinoids. Our data on cisplatin, paclitaxel, adriamycin, UV radiation, and H_2_O_2 _in Figure 2 indicate a lack of major cross-resistance. All of these agents have been described to act, among other pathways, by increasing endogenous Cer [44-48]. Therefore, our results could be interpreted as a lack of cross-resistance against Cer-increasing agents. The possible implication of Cer must be taken with caution because a) all of these insults have been described to increase ROS production [49-52], b) most studies have been performed by [^3^H]palmitic acid labeling or DAG kinase plus TLC separation, which do not distinguish between Cer and dhCer, and c) we recently described that oxidative stress leads to dhCer, and not Cer, accumulation [53].

## Conclusions

In conclusion, development of the first 4-HPR-resistant ALL cell lines has revealed that observed alterations in the endogenous SL pattern are reversible and can be dissociated from the resistance phenotype in leukemia. Moreover, sensitivity of 4-HPR-resistant ALL cells to SL modulating treatments was comparable to the sensitivity observed in parental sensitive leukemia cells. We have also shown that 4-HPR resistance does not induce cross-resistance to other clinically applied drugs, providing feasible solutions for 4-HPR-resistant ALL leukemias.

## Competing interests

The authors declare that they have no competing interests.

## Authors' contributions

The experiments were carried out by AAp and JIB. AAs and AAp conceived the study, and all authors participated in the study design. The draft was written by AAp, JIB, and MDB, and the intellectual content revised by GPY, YAH, and AAs. All authors read and approved the final manuscript.

## Pre-publication history

The pre-publication history for this paper can be accessed here:

http://www.biomedcentral.com/1471-2407/11/477/prepub

## Supplementary Material

Additional file 1 **Comparative cellular proliferation of resistant cells after drug withdrawal**. CCRF-CEM, R10, and R10 long WD cells (1.5 × 10^6 ^cells/cell line) were incubated for 15 min in CFSE-containing pre-warmed PBS (0.36 μM, CO_2 _incubator). The PBS was replaced by FBS-supplemented culture medium for 30 min. A total of 0.5 × 10^6 ^cells/cell line were fixed with 2% paraformaldehyde-containing PBS and stored at 4°C to use them as parental (P) cells. A similar amount of cells was fixed after 24 h and 48 h incubation. All samples were washed with PBS prior to measuring fluorescence (excitation 485 nm and emission 530 nm) by flow cytometry (Beckman Coulter Gallios; General Research Services SGIker of the UPV/EHU (http://www.ikerkuntza.ehu.es/p273-sgikerhm/en/). Generations (G) were determined using the ModFit LT™ software. Click here for file

Additional file 2 **Toxicity profiles after combination of 4-HPR with other SL modulators**. CCRF-CEM cells were co-treated with 4-HPR (1-3 μM) and DHS (unnatural dhSph analogue) or PPMP (glucosylceramide synthase inhibitor) for 24 h and viability estimated by metabolic activity (XTT assay). Data are average ± SD of at least three independent experiments performed in quadruplicate (n ≥ 12); *P < 0.05,**P < 0.01; ANOVA plus Tamhane or Bonferroni post-hoc test. Click here for file

Additional file 3 **Effect of 4-HPR and other SL modulators on the cellular SL pattern: TOTALdhCer and TOTALCer**. 4-HPR sensitive ALL cells (CCRF-CEM and Jurkat) were incubated for 2 h or 6 h with 4-HPR (1 μM), PPMP (10 μM), DHS (5 μM), and/or SAF (4 μM) and the SL level determined. Complex treatments refer to 4-HPR+PPMP with either DHS or SAF. Data are average ± SD of an experiment performed in duplicate. Click here for file

Additional file 4 **Effect of 4-HPR and other SL modulators on the cellular SL pattern: TOTAL GluCer and TOTAL LactCer**. 4-HPR-sensitive ALL cells (CCRF-CEM and Jurkat) were incubated for 2 h or 6 h with 4-HPR (1 μM), PPMP (10 μM), DHS (5 μM), and/or SAF (4 μM) and SL levels determined. Complex treatments refer to 4-HPR+PPMP with either DHS or SAF. Data are average ± SD of an experiment performed in duplicate. Click here for file

Additional file 5 **Effect of 4-HPR and other SL modulators on the cellular SL pattern: dhSph and Sph**. 4-HPR-sensitive ALL cells (CCRF-CEM and Jurkat) were incubated for 2 h or 6 h with 4-HPR (1 μM), PPMP (10 μM), DHS (5 μM), and/or SAF (4 μM) and SL levels determined. Complex treatments refer to 4-HPR+PPMP with either DHS or SAF. Data are average ± SD of an experiment performed in duplicate. Click here for file
