# Supplementary figures and images for: Evaluation of bioactive sphingolipids in 4-HPR-resistant leukemia cells
Source: BMC Cancer. 2011 Nov 7;11:477. doi: 10.1186/1471-2407-11-477 (PMC3218121; doi:10.1186/1471-2407-11-477)

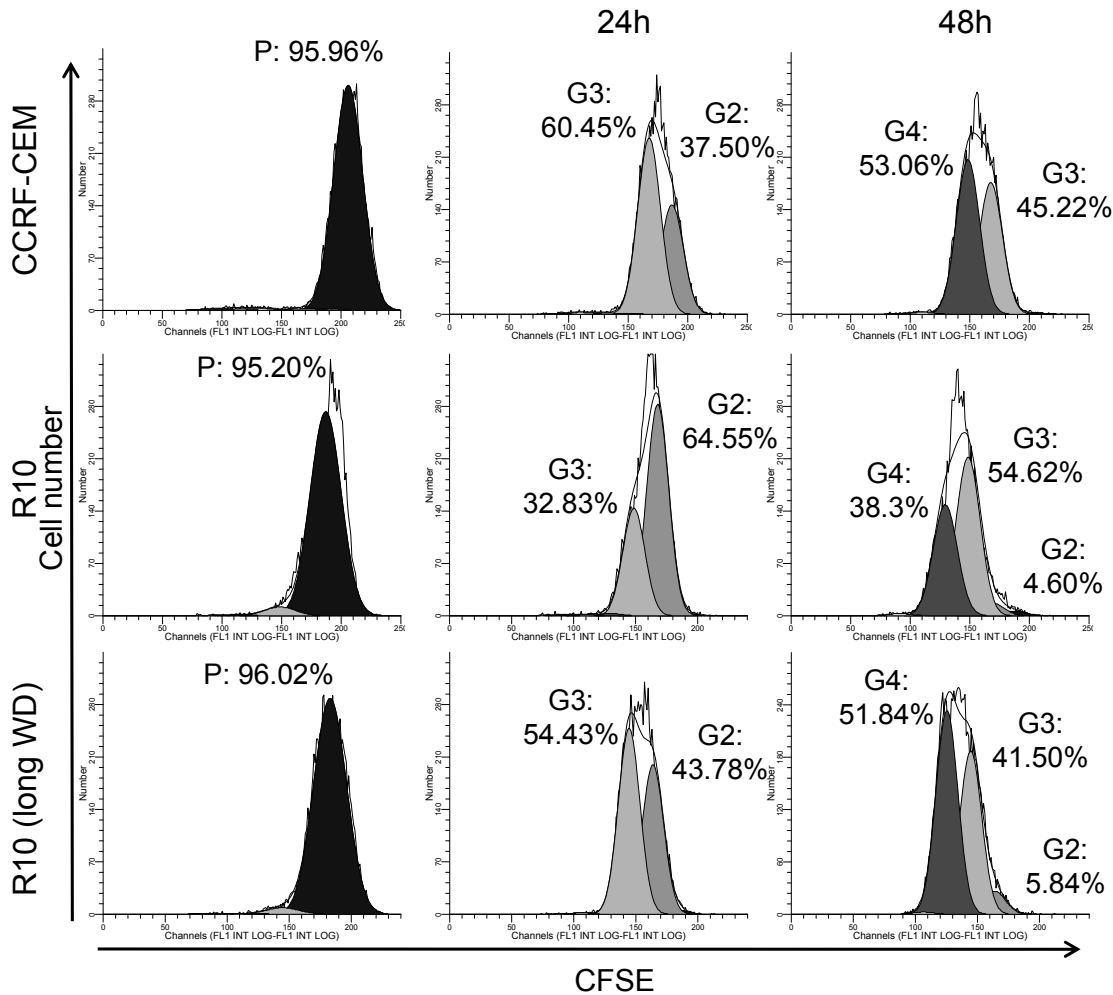

Supplement: Additional file 1 — Comparative cellular proliferation of resistant cells after drug withdrawal. CCRF-CEM, R10, and R10 long WD cells (1.5 × 10^6^ cells/cell line) were incubated for 15 min in CFSE-containing pre-warmed PBS (0.36 μM, CO2 incubator). The PBS was replaced by FBS-supplemented culture medium for 30 min. A total of 0.5 × 10^6^ cells/cell line were fixed with 2% paraformaldehyde-containing PBS and stored at 4°C to use them as parental (P) cells. A similar amount of cells was fixed after 24 h and 48 h incubation. All samples were washed with PBS prior to measuring fluorescence (excitation 485 nm and emission 530 nm) by flow cytometry (Beckman Coulter Gallios; General Research Services SGIker of the UPV/EHU (http://www.ikerkuntza.ehu.es/p273-sgikerhm/en/). Generations (G) were determined using the ModFit LT™ software. [file 1471-2407-11-477-S1.PDF]

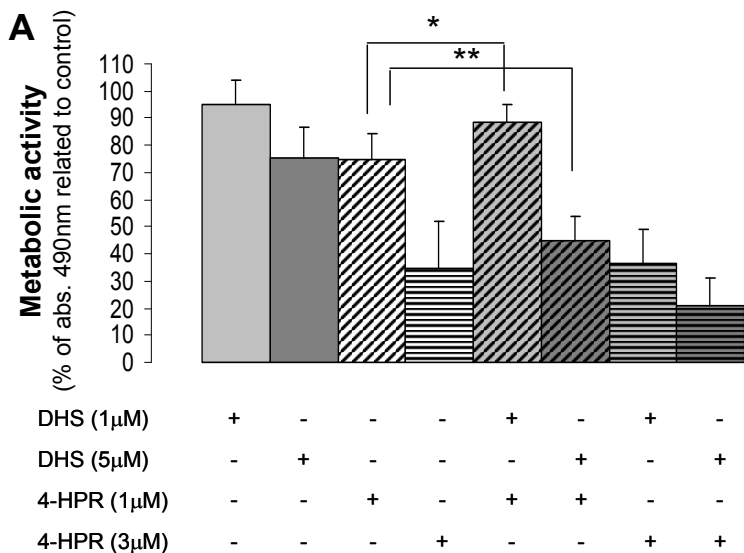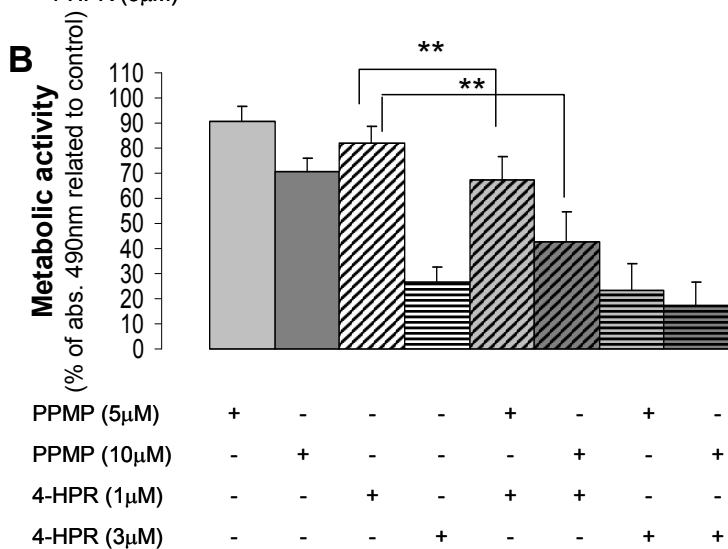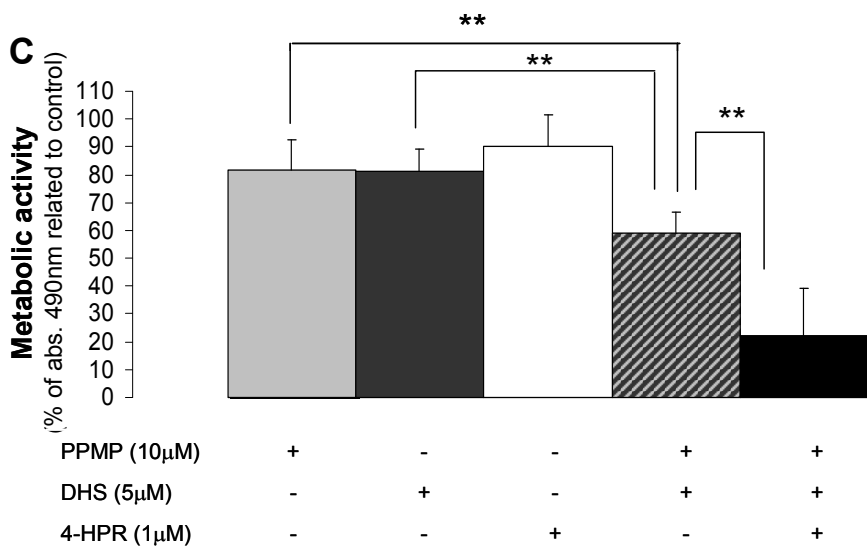

Supplement: Additional file 2 — Toxicity profiles after combination of 4-HPR with other SL modulators. CCRF-CEM cells were co-treated with 4-HPR (1-3 μM) and DHS (unnatural dhSph analogue) or PPMP (glucosylceramide synthase inhibitor) for 24 h and viability estimated by metabolic activity (XTT assay). Data are average ± SD of at least three independent experiments performed in quadruplicate (n ≥ 12); *P < 0.05,**P < 0.01; ANOVA plus Tamhane or Bonferroni post-hoc test. [file 1471-2407-11-477-S2.PDF]

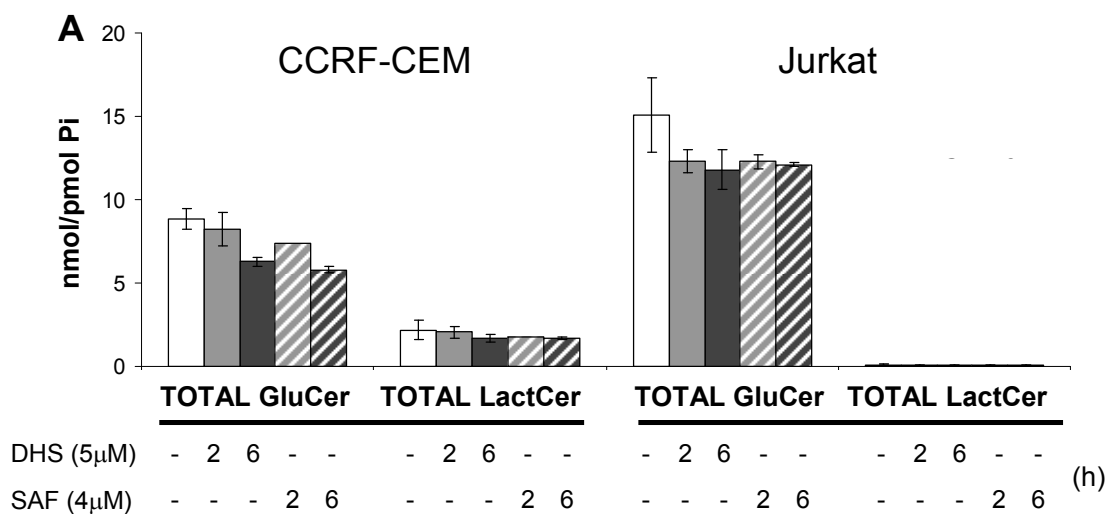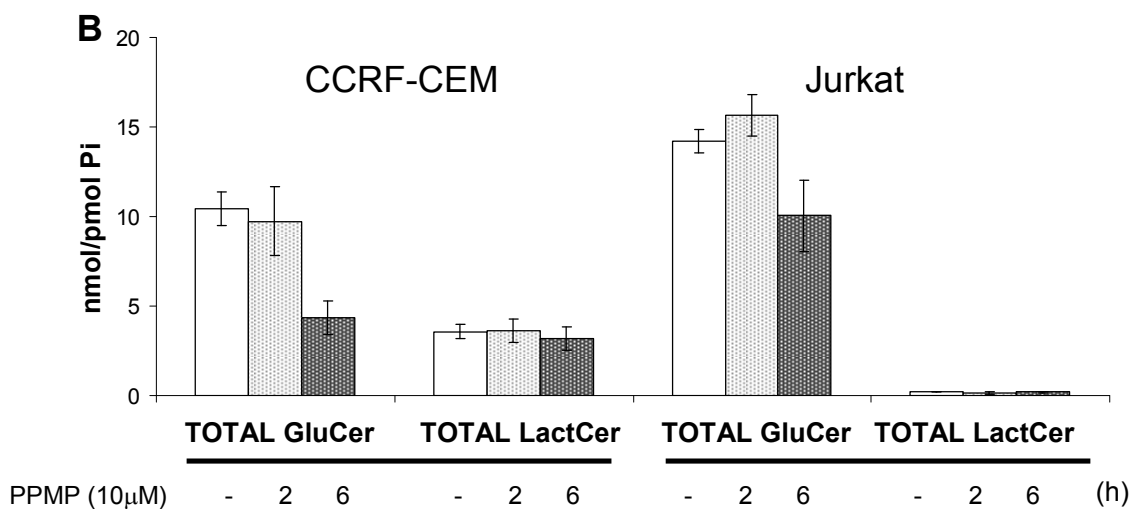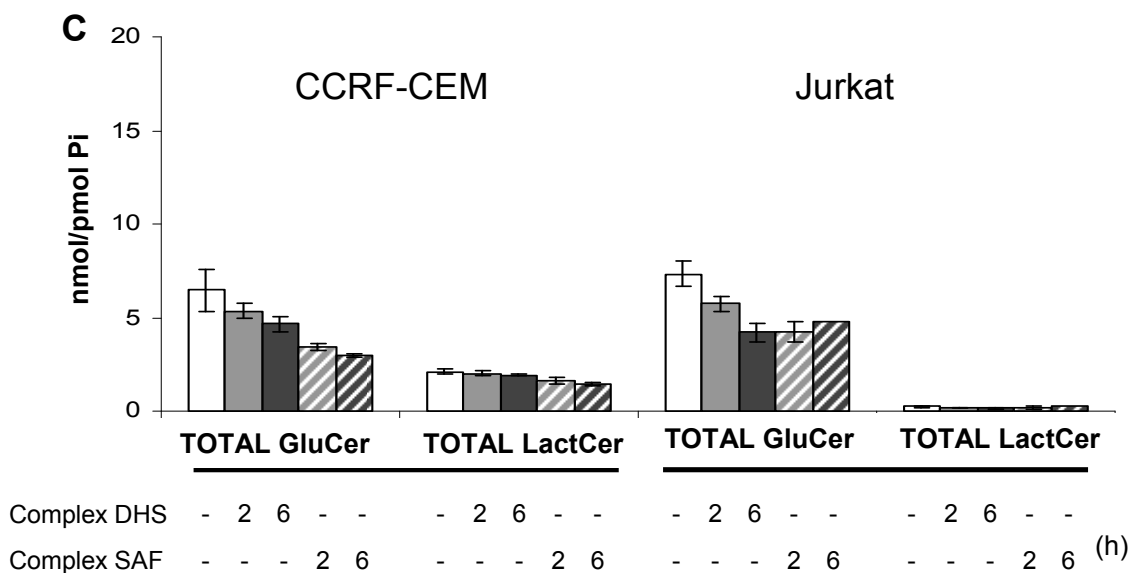

Supplement: Additional file 4 — Effect of 4-HPR and other SL modulators on the cellular SL pattern: TOTAL GluCer and TOTAL LactCer. 4-HPR-sensitive ALL cells (CCRF-CEM and Jurkat) were incubated for 2 h or 6 h with 4-HPR (1 μM), PPMP (10 μM), DHS (5 μM), and/or SAF (4 μM) and SL levels determined. Complex treatments refer to 4-HPR+PPMP with either DHS or SAF. Data are average ± SD of an experiment performed in duplicate. [file 1471-2407-11-477-S4.PDF]

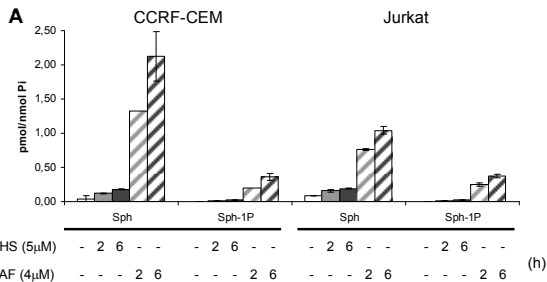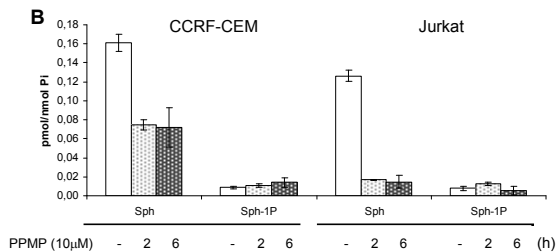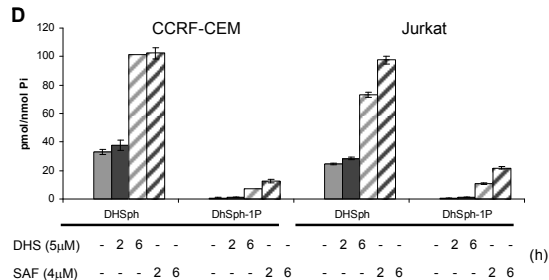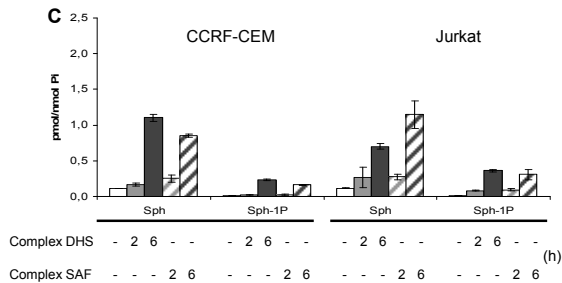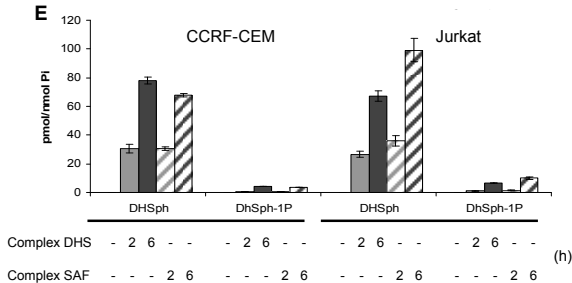

Supplement: Additional file 5 — Effect of 4-HPR and other SL modulators on the cellular SL pattern: dhSph and Sph. 4-HPR-sensitive ALL cells (CCRF-CEM and Jurkat) were incubated for 2 h or 6 h with 4-HPR (1 μM), PPMP (10 μM), DHS (5 μM), and/or SAF (4 μM) and SL levels determined. Complex treatments refer to 4-HPR+PPMP with either DHS or SAF. Data are average ± SD of an experiment performed in duplicate. [file 1471-2407-11-477-S5.PDF]
