# Supplementary material for: Evaluation of bioactive sphingolipids in 4-HPR-resistant leukemia cells
Source: BMC Cancer. 2011 Nov 7;11:477. doi: 10.1186/1471-2407-11-477 (PMC3218121; doi:10.1186/1471-2407-11-477)
Supplement: Additional file 3 — Effect of 4-HPR and other SL modulators on the cellular SL pattern: TOTALdhCer and TOTALCer. 4-HPR sensitive ALL cells (CCRF-CEM and Jurkat) were incubated for 2 h or 6 h with 4-HPR (1 μM), PPMP (10 μM), DHS (5 μM), and/or SAF (4 μM) and the SL level determined. Complex treatments refer to 4-HPR+PPMP with either DHS or SAF. Data are average ± SD of an experiment performed in duplicate. [file 1471-2407-11-477-S3.PDF]

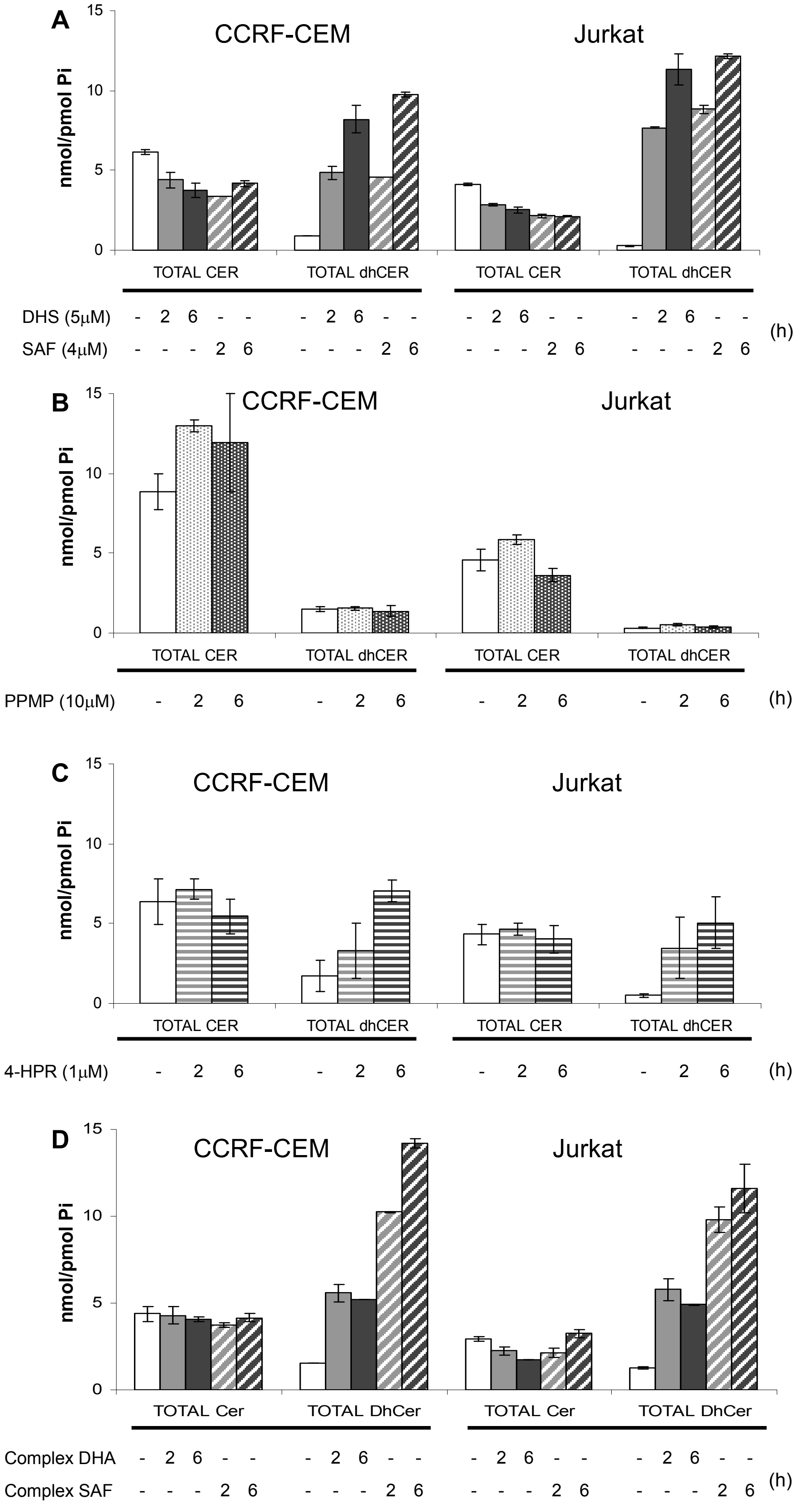
A
CCRF-CEM
Jurkat
nmol/pmol Pi
TOTAL CER
TOTAL dhCER
DHS (5μM)
SAF (4μM)
(h)
B
PPMP (10μM)
C
4-HPR (1μM)
D
TOTAL Cer
TOTAL DhCer
Complex DHA
Complex SAF
